# Isolation and purification of antibacterial lipopeptides from Bacillus velezensis YA215 isolated from sea mangroves

**DOI:** 10.3389/fnut.2022.1064764

**Published:** 2022-11-24

**Authors:** FuTian Yu, YuanYuan Shen, YaLi Qin, YiYang Pang, HeLiang Fan, JingJing Peng, XiaoDong Pei, XiaoLing Liu

**Affiliations:** College of Light Industry and Food Engineering, Guangxi University, Nanning, China

**Keywords:** Bacillus velezensis, antimicrobial activity, lipopeptides, surfactin, mechanism

## Abstract

The increasing burden and health risks of antimicrobial resistance (AMR) pose a great threat to society overall. Lipopeptides exhibit great potential as novel and safe alternatives to traditional antibiotics. In this study, the strain YA215, which was isolated from the mangrove area in Beibu Gulf, Guangxi, China, was identified as Bacillus velezensis. Then, YA215 lipopeptide extracts (YA215LE) from B. velezensis was found to exhibit a wide spectrum of antibacterial and antifungal activities. Additionally, YA215LE was identified and found to contain three groups of lipopeptides (surfactin, iturin, and fengycin). Furthermore, one separation fraction (BVYA1) with significant antibacterial activity was obtained. Additionally, liquid chromatography tandem mass spectrometry (LC-MS/MS) analysis of BVYA1 showed three molecular ion peaks ([M + H]^+^: m/z 980.62; 994.66; 1008.66) corresponding to conventional surfactin homologs. By MS/MS analysis, BVYA1 was identified as sufactin with the precise amino acid sequence Glu–Leu/Ile–Leu–Val–Asp–Leu–Leu/Ile and hydroxyl fatty acids with 11–13 carbons. [M + H]^+^ at m/z 980.62 was detected for the first time in B. velezensis, which demonstrates that the strain corresponds to a new surfactin variant. In particular, BVYA1 showed antibacterial activity with the minimum inhibitory concentration (MIC) values of 7.5–15 μg/ml. Finally, the preliminary mechanism of inhibiting *E. coli* treated with BVYA1 showed that BVYA1 effectively permeabilized the cytoplasmic membrane and disrupted the morphology of targeted bacterial cells. In conclusion, this study suggests that the YA215LE from B. velezensis YA215 might be a potential candidate for a bactericide.

## Introduction

The increasing the burden and health risks of antimicrobial resistance (AMR) pose a great threat to society as a whole. It is estimated that approximately 1,000,000 people die each year worldwide from AMR-related diseases (1, 2). Key factors contributing to the emergence of antibiotic resistance in microorganisms include the unregulated use of antibiotics, misuse of antibiotics, inappropriate regulation of antibiotics, and lack of research and discovery of new antibiotics (3). With the urgent necessity of protecting the environmental and achieving green and sustainable development, the search for a new and safe alternative to traditional antibiotics has become an urgent research problem that must be solved. Antimicrobial peptides, phage, nanoparticles, and photodynamic therapy are promising novel alternatives that are currently being investigated to counter the adverse effects caused by AMR (4, 5). Among them, antimicrobial peptides exhibit potential to be developed as alternatives to traditional antibiotics, as antimicrobial peptides perform rapid and specific actions and exhibit high diversity in their structural and functional activities (6). Among antimicrobial peptides, lipopeptides have been extensively studied as potential antibacterial and antifungal agents (7).

Lipopeptides were produced by different species of microbial genera, which included Bacillus, Streptomyces, and Pseudomonas (8, 9). However, the genus that was found to produce the most lipopeptides was Bacillus, and the earliest antimicrobial lipopeptides were also produced by Bacillus. Lipopeptides produced by Bacillus sp. were catalyzed by non-ribosomal peptide synthases (NRPSs) (8). The structures of these lipopeptides exhibit a remarkable diversity in the type and sequence of amino acid residues, the properties of peptide cyclization, and the properties, length, and branching of fatty acid chains (8). Approximately 5–8% of the Bacillus total genome was dedicated to synthesizing bioactive secondary metabolites, such as peptides and lipopeptides, polyketides, bacteriocins, and siderophores (10). The main Bacillus species that produce lipopeptides were B. amyloliquefaciens, B. subtilis, B. velezensis, and B. licheniformis. (11). Lipopeptides can be divided into three families (surfactin, iturin, and fengycin) (12). The peptide chains of both surfactin and iturin were partially composed of cyclic heptapeptides, and their fatty chains were partially composed of β-hydroxy fatty acid chain between the C13 and C17 carbons and β-amino fatty acid chain between the C15 and C18 carbons, respectively; and the peptide chain of fengycin was composed of decapeptides and the fatty chain was composed of β-hydroxy fatty acid chains between the C12 and C19 carbons (13). Since these Bacillus have different structures and properties, the lipopeptides produced also exhibit different antimicrobial activities; for example, the surfactin families are responsible for antibacterial activities but have limited antifungal activity (14); the iturin families are associated with strong antifungal activity (15, 16); and the fengycin families, which are less hemolytic than either the surfactin families or the the iturin families, exhibit a strong antifungal capability (17–19). Lipopeptides have a unique chemical composition and amphiphilic ring structure, which make their structure stable and can maintain high activity under some unfavorable conditions, such as high temperature, ultraviolet light and different pH values and proteases (12, 20). Apart from that, these lipopeptides exhibit unique properties; for example, these lipopeptides are easily biodegradable, eco-friendly, non-polluting, and safety (21, 22). Due to these advantages, lipopeptides are becoming increasingly vital in applications such as pharmaceuticals, agriculture and food preservation (21, 23). These results show that lipopeptides could potentially be developed as alternatives to traditional antibiotics.

Therefore, in this study, antibacterial lipopeptides were isolated and identified as novel alternatives to traditional antibiotics; specifically, this study was designed to isolate, purify, and characterize antibacterial lipopeptides from the YA215 strain, which was isolated from the mangrove area in Beibu Gulf, Guangxi, China. First, the YA215 strain, which was isolated from the mangrove area in Beibu Gulf, Guangxi, China, was identified as B. velezensis based on morphological and 16S rRNA gene sequences. Second, the antimicrobial activity of YA215LE, which were obtained by the combining hydrochloric acid precipitation and methanol extraction, was evaluated by the agar well diffusion method. In addition, the composition of YA215LE was identified using liquid chromatography tandem mass spectrometry (LC-MS/MS) analysis. Furthermore, YA215LE was isolated and purified by gel column chromatography and semiprepared reverse-phase high-performance liquid chromatography (RP-HPLC), and one separation fraction (BVYA1) with significant antibacterial activity was obtained. BVYA1 was identified by MS/MS analysis, its MIC was determined. Finally, the preliminary mechanism of inhibition of *E. coli* treated with BVYA1 was derived by scanning electron microscopy (SEM) and calcein-AM/PI double-staining assays. The results will provide valuable information for further screening a novel alternative to traditional antibiotics from the B. velezensis YA215 strain.

## Materials and methods

### Materials

Lysogeny broth (LB) medium, potato dextrose agar (PDA) medium and agar was obtained from Guangdong Huan Kai Biotechnology Co., Ltd. (Guangzhou, China). Trifluoroacetic acid (TFA), methanol and acetonitrile were chromatography grade, hydrochloric acid, disodium hydrogen phosphate, sodium dihydrogen phosphate, glutaraldehyde, ethanol, and sodium hydroxide were analytical pure and these were purchased from Shanghai Macklin Biochemical Co., Ltd. (Shanghai, China).

### Microbial strains

The YA215 strain, which was isolated by our research team from the mangrove area in Beibu Gulf, Guangxi, China, was used in the current study (24, 25). *Escherichia coli* (CGMCC 112252), *Bacillus nanotuberculosis*, *Bacillus subtilis CGMCC 1.14985*, *Bacillus subtilis CGMCC 1.15792*, *Staphylococcus aureus CGMCC26003, Salmonella typhimurium CGMCC11190*, *Shigella flexneri CGMCC110599*, and *Rhizopus stolonifer CGMCC3.31* were obtained from the National Center for Medical Culture Collections (CMCC, Beijing, China), and *Bacillus licheniformis*, *Bacillus cereus, Fusarium oxysporum f.sp.cubense*, and *Penicillium expansum CICC 40658* were stored in a 40% glycerol solution at –80^°^C in this team.

### Identification of the YA215 strains

YA215 strain was grown on LB agar medium for 24 h at 37^°^C, and then the colony morphology of the YA215 strain was observed, in addition a single colony was picked for Gram staining. Subsequently, strain confirmation was performed by 16S rRNA gene amplification with the following regular pair of forward and reverse primers; 27F (5′-GGTTACCTTGTTACGACTT-3′) and 1492R (5′-AGAGTTTGATTTGATCCTGGCTAG-3′). The amplification was carried out in a polymerase chain reaction (PCR) instrument (TC-512, Biometra Company, Germany) programmed using the following protocol: 94^°^C for 5 min; 35 cycles of 94^°^C for 30 s, 64^°^C for 1 min, and 72^°^C for 2 min and then a final elongation step of 72^°^C for 5 min. The analyzed PCR products were sent to Sangon Biotech Co., Ltd. (Shanghai, China) for sequencing. Then, the 16S rDNA sequences were compared with the GenBank database using BLAST in NCBI data, along with homology analysis, and a phylogenetic tree was constructed using MEGA 7.0 software.

### Lipopeptide extraction

YA215 strain was activated on LB agar plates for 24 h, and then a single colony was picked and inoculated into 100 ml LB broth and incubated at 37^°^C and 220 rpm for 12 h. Then, a 4% (v/v) inoculum was fermented in pill bottles at 37^°^C and 220 rpm for 48 h. The cell-free supernatant was obtained after centrifugation at 4^°^C and 10,000 g. The YA215LE was obtained by a combination of acid precipitation and methanol extraction method (26). Briefly, The pH of the cell-free supernatant was adjusted to 2.0 with 6 M hydrochloric acid solution, then it was left to stand overnight at 4^°^C. The precipitate was then collected by centrifugation for 20 min at 4^°^C at 10,000 pitate was then collects then washed twice with distilled water at pH 2.0. Afterward, the precipitate was resuspended in distilled water and the pH was adjusted to 7.0, and then it was freeze-dried. The dried sample was then extracted with methanol and the methanol soluble fraction was dried with a rotary vacuum evaporator at 45^°^C. After removal of methanol, trace amounts of distilled water were added to dissolve YA215LE. The solution was then freeze-dried again and weighed for quantification.

### Determination of the antimicrobial activity of YA215 lipopeptide extracts

The antibacterial activity of YA215LE was evaluated by the agar well diffusion method with minor modifications (27). Briefly, indicator strains obtained from logarithmic growth phase culture at a final concentration of 10^6^ cfu/ml were added to LB solid medium that was cooled below 50^°^C. Then, the LB solid medium was poured into the Petri dishes. After the medium solidified, holes with a diameter of 6 mm were punched on the medium with a sterile punch, and then 100 μ of YA215LE solution was added to the holes. After that, the Petri dishes were left to stand overnight at 37^°^C. The antibacterial activity of YA215LE was determined by measuring the diameter of the inhibition zone around the holes.

Antifungal activity was evaluated by the agar well diffusion method with minor modifications (28). Mycelial blocks (6 mm in diameter) were obtained from the central part of the 7 days-old fungal indicator strain and then placed onto the center of a culture dish loaded with fresh sterile PDA medium. After 4 days of incubation at 28^°^C, the medium was perforated (5 mm diameter) with a sterile punch at a distance of 2 cm from the mycelium, and 100 μl of YA215LE solution was added to the holes. The culture dish was continued to be incubated at 28^°^C for 4 days, and then the diameter of the inhibition zone was measured.

### Purification of YA215 lipopeptide extracts

The YA215LE obtained from the fermentation broth of the YA215 strain was first was purified on a Sephadex G-25 (Shanghai Xiamei Biochemical Technology Development Co., Ltd., China) gel filtration column (1.6 × 100 cm). The samples were eluted with phosphate buffered saline (PBS) (20 mM, pH 7.0) at a flow rate of 0.3 ml/min, and the absorbance was monitored at 280 nm. An automatic collection device was used to collect the eluent for 10 min per tube. The antibacterial activity of each canal was determined by using the agar well diffusion method as described before. The active eluent collected was further purified by semipreparative RP-HPLC using the method previously described (29) with modifications. The 100 μl sample solution was injected into the RP-HPLC (Waters Company, MA, USA) through a semipreparative column (XBridge™BEH130 C18, 10 × 250 mm). The mobile phase consisted of eluent A (0.1% v/v TFA) in ultrapure water and eluent B (methanol with 0.1% v/v TFA). The sample was separated using the following gradient: 0–50 min: 5–100% B; 50–51 min: 100–5% B; 51–60 min with 5% B. The flow rate of the mobile phase was set to 2 ml/min. The UV detector detected the elution peak at 280 nm and the same elution peak fractions were collected together and they were freeze-dried to detect their antimicrobial activity.

The purity of the elution peak with the highest antibacterial activity obtained by semipreparative RP-HPLC was measured by an analytical RP-HPLC system equipped with a C18 analytical column (4.6 × 250 mm, 100 Å, 5 μm, Waters, USA) with the same elution procedure. The injection volume was 20 μl, and the flow rate of the mobile phase was set to 0.5 ml/min.

### Identification of the antimicrobial active fraction isolated from YA215 lipopeptide extracts

The identification of the eluted fraction with antibacterial activity obtained by RP-HPLC was performed by LC-MS/MS analysis as described previously (30, 31). Mass spectrometry detection equipment with C18 analytical column (Acclaim PepMapTM RSLC, C18, 100 Å, 2, 50 μm analytical columnivity obtained by R) was used to identify antimicrobial active compounds. The mobile phases consisted of eluent A (2% acetonitrile, 0.1% formic acid, 97.9% water) and eluent B (98% acetonitrile, 0.1% formic acid, 1.9% water). The column was eluted by the following gradient: 0–5 min with 2–12% B; 5–30 min with 12–20% B; 30–43 min with 20–32% B; 43–48 min with 32–98% B; 48–58 min with 98% B, and the flow rate of the mobile phase was set to 300 nl/min. The mass spectrometry measurement conditions were: positive ion scan, voltage 1.9 kV, temperature 270°C, energy 29% higher energy collision induced dissociation (HCD), and scan range m/z 300–1,800.

### Determination of the minimum inhibitory concentration of the antimicrobial active fraction isolated from YA215 lipopeptide extracts

The MIC of BVYA1 was determined by the 2-fold dilution method using a sterile 96-well plate (32). 100 μl of 30 μg/ml BVYA1 was added to wells containing 100 μl of fresh LB broth medium and serially diluted. Then 100 μl of the indicator bacteria (10^8^ cfu/ml) was added to each well. The MIC value of BVYA1 was determined as the lowest concentration at which growth did not occur. The MIC was investigated in duplicate wells and triplicate experiments.

### Scanning electron microscopy analysis

The indicator strain *E. coli* in the log phase of growth was incubated with BVYA1 (2 × MIC) at 37^°^C for 2 h. The bacterial cells treated with PBS (pH 7.4) served as a control. Bacterial cells were collected by centrifugation at 5,000 rpm for 15 min. Bacterial cells were washed three times with 20 mM PBS (pH 7.4). The bacterial cells were then fixed with 2.5% glutaraldehyde at 4^°^C overnight. After that, the bacterial cells were washed three times with 20 mM PBS. The bacterial cells were then dehydrated in a series of graded ethanol (30–100%) for 10 min at each concentration. Afterward, the bacterial cells were freeze-dried and sputtered for gold plating prior to the assay (33). The prepared samples were examined using SEM (FEI Quattro S, America) at an accelerating voltage of 8.0 kV.

### Measuring the activity of bacterial membrane disruption

The effect of BVYA1 on bacterial membrane integrity was measured using a calcein-AM/propidium iodide (PI) double staining kit (Beijing Solarbio Science and Technology Co., Ltd) performed according to a previously described method (34) with some modifications. Log phase *E. coli* cells were suspended in 100 μl of 10 mM PBS (pH 7.4) at a concentration of 2 × 10^6^ cells/ml and then treated with 50 μl BVYA1 (2 × MIC) for 30 min at 37^°^C. Cells were then collected by centrifugation at 8,000 × g for 10 min and resuspended in 100 μl of PBS containing 0.1 μl of calcein-AM stock solution. After incubation at 37^°^C for 25 min, 5 μl of PI stock solution was added to the reaction system. After continued incubation at 37^°^C for 5 min, the cells were washed three times with PBS and then fixed on microscope glass slides by air-drying. Imaging was performed using a fluorescence microscope (DM4B; Leica, Germany). The control sample was treated with PBS, and observed under the same conditions.

### Statistical analysis

Each assay was performed in triplicate. The experimental data are presented as the mean ± standard deviation.

## Results

### Identification of the YA215 strain

**FIGURE 1:**
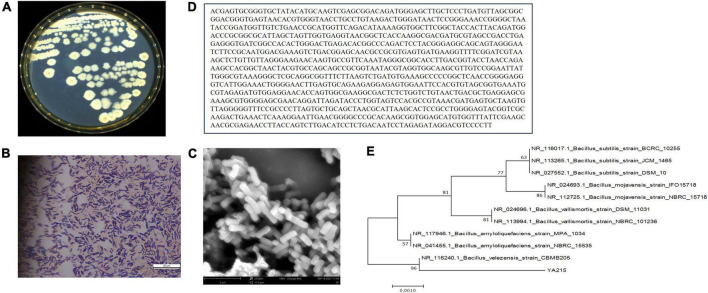
Identification of the YA215 strain. **(A)** Colonial morphology of the YA215 strain. **(B)** Gram staining of the YA215 strain. **(C)** Electron microscopic picture of the YA215 strain. **(D)** Sequence of the 16S DNA gene of the YA215 strain. **(E)** Neighbor-joining phylogenetic tree of the YA215 strain.

To classify the YA215 strain more differently at the species level, observation of its cellular morphology and homologous analysis of its 16S rDNA sequences were conducted. YA215 strain was found to be yellowish and round with irregular colony edges after 24 h incubation at 37^°^C (Figure 1A). Through Gram staining and SEM, the cells of strain YA215 were observed to be typically rod-shaped with blunt ends under Gram staining and SEM (Figures 1B,C). At the molecular level, PCR amplification of the 16S rRNA gene of the YA215 strain was sequenced and submitted to GenBank. BLAST results indicated that the constructed phylogenetic tree revealed 96% similarity between the YA215 strain and the Bacillus velezensis CBMB205 strain (Figures 1D,E). The strain YA215 was thus identified as B. velezensis and was named as B. velezensis YA215.

### Antimicrobial activity of the lipopeptide extracts

**FIGURE 2:**
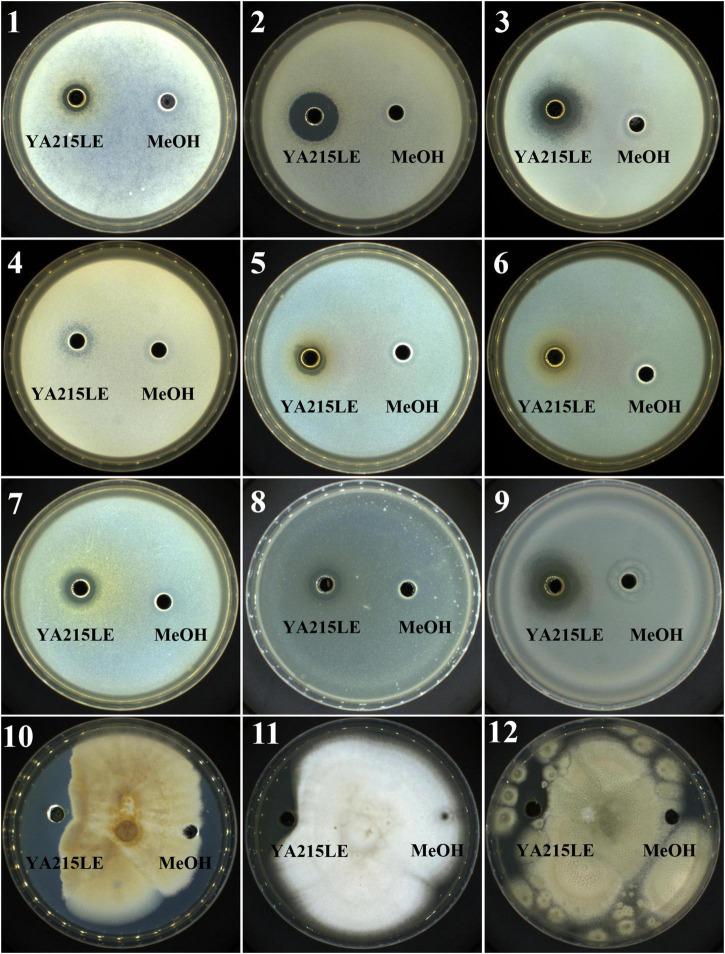
Demonstration of the inhibitory effects of YA215 lipopeptide extracts (YA215LE) against phytopathogenic bacteria and fungi (panels 1-12). (1) *Bacillus licheniformis*, (2) *Bacillus cereus*, (3) *Staphylococcus aureus CGMCC26003*, (4) *Bacillus nanotuberculosis*, (5) *Bacillus subtilis CGMCC1.14985*, (6) *Bacillus subtilis CGMCC1.15792*, (7) *Escherichia coli CGMCC44102*, (8) *Salmonella typhimurium CGMCC11190*, (9) *Shigella flexneri CGMCC110599*, (10) *Fusarium oxysporum f.sp.cubense*, (11) *Rhizopus stolonifer CGMCC3.31*, (12) *Penicillium expansum CICC 40658*.

The antimicrobial activity of the YA215LE was evaluated by the agar well diffusion method. The YA215LE exhibited a wide spectrum of antibacterial and antifungal activities. The YA215LE exhibited a good inhibitory effect on gram-positive bacteria, such as *Bacillus licheniformis*, *Bacillus cereus*, *Staphylococcus aureus CGMCC26003*, *Bacillus nanotuberculosis*, *Bacillus subtilis CGMCC1.14985*, and *Bacillus subtilis CGMCC1.15792*, and on gram-negative bacteria, such as *Escherichia coli CGMCC44102*, *Salmonella typhimurium CGMCC11190*, and *Shigella flexneri CGMCC110599*. In the antifungal activity assay, YA215LE also exhibited strong inhibitory effects on *Fusarium oxysporum f.sp.cubense*, *Rhizopus stolonifer CGMCC3.31*, and *Penicillium expansum CICC 40658* (Figure 2). These results suggest that YA215LE exhibits broad-spectrum antimicrobial effects.

### Identification of the composition of YA215 lipopeptide extracts

**FIGURE 3:**
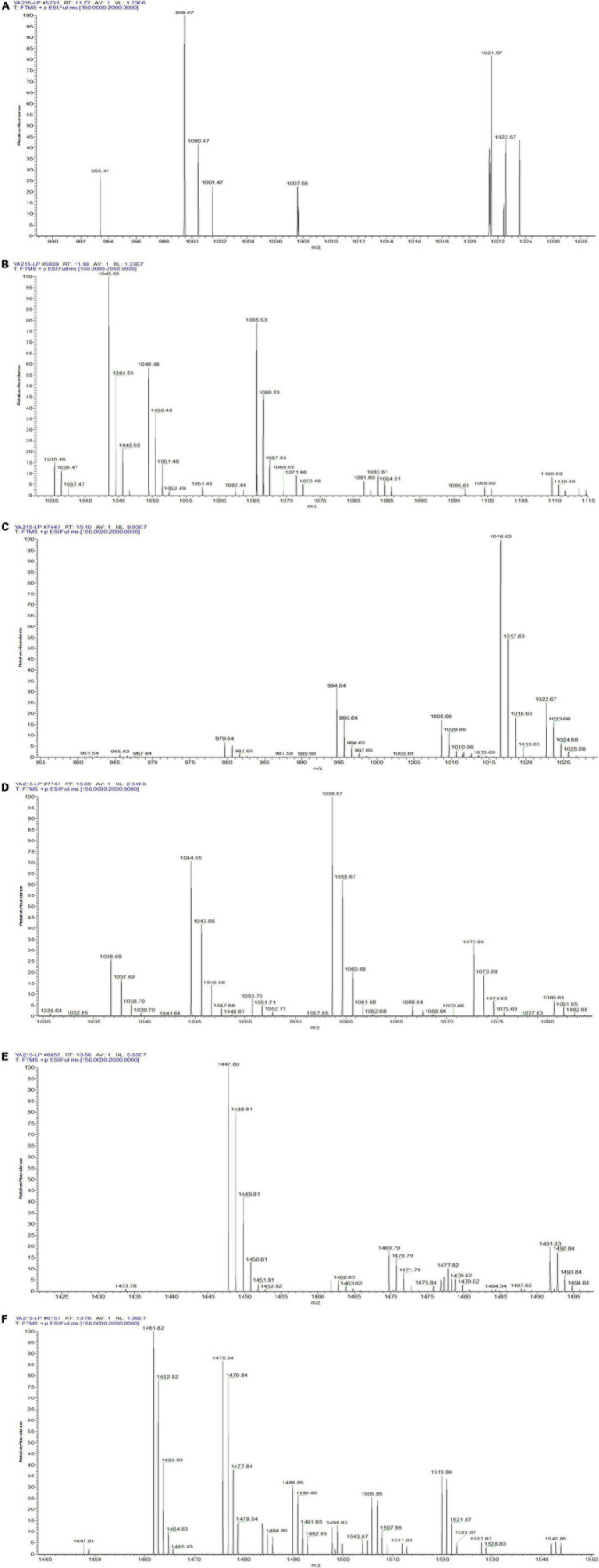
LC–MS spectrum of the composition of YA215 lipopeptide extracts (YA215LE). **(A)** MS spectrum of the molecular ion peaks [M + H]^+^ at 993.41, 1007.59 and 1021.57, **(B)** MS spectrum of the molecular ion peaks [M + H]^+^ from 1035.46 to1099.60, **(C)** MS spectrum of the molecular ion peaks from 994.64 to 1022.67, **(D)** MS spectrum of the molecular ion peaks from 1030.64 to 1072.68, **(E)** MS spectrum of fengycin A analogs with the molecular ion peaks from 1449.81 to 1519.86, **(F)** MS spectrum of fengycin B analogs with the molecular ion peaks from 1433.78 to 1489.85.

Identification of the composition of YA215LE was assessed using liquid chromatography-mass spectrometry (LC-MS) analysis. The strain produced molecules with m/z corresponding to cyclic lipopeptides from different families that were previously described in the Bacillus genera, namely, surfactins, iturins, and fengycins (35). Iturin homologs with molecular ion peaks [M + H]^+^ of 993.41, 1007.59, 1021.57, 1035.46, 1049.48, and 1063.44 were detected, corresponding to C12 to C17 hydroxy fatty acid chains (Figures 3A,B). Mycosubtilin homologs belonging to the iturin family with molecular ion peaks [M + H]^+^ 1043.55, 1057.45, 1071.46, 1085.59, and 1099.60 were also detected, corresponding to C14 to C18 hydroxy fatty acid chains (Figure 3B). For the molecules biosynthesized from the YA215 strain, the m/z values were attributed to analogs of surfactin. These analogs contain various fatty acid tails, i.e., C12 (m/z 994.64 [M + H]^+^, m/z 1016.62 [M + Na]^+^), C13 (m/z 1008.66 [M + H]^+^, m/z 1030.64 [M + Na]^+^), C14 (m/z 1022.67 [M + H]^+^, m/z 1044.65 [M + Na]^+^), C15 (m/z 1036.69 [M + H]^+^, m/z 1058.67 [M + Na]^+^), and C16 (m/z 1050.70 [M + H]^+^, 1072.68 [M + Na]^+^) (Figures 3C,D). These m/z values could also correspond to many other surfactin analogs that contain amino acid modifications at different locations on the peptide chain. Finally, fengycin A analogs with different possible fatty acid chains were detected, i.e., C15 (m/z 1449.81 [M + H]^+^), C16 (m/z 1463.82 [M + H]^+^), C17 (m/z 1477.82 [M + H]^+^), C18 (m/z 1491.83 [M + H]^+^), C19 (m/z 1505.85 [M + H]^+^), and C20 (m/z 1519.86 [M + H]^+^) (Figures 3E,F). Fengycin B analogs with different possible fatty acid chains were also detected, i.e., C14 (m/z 1433.78 [M + H]^+^), C15 (m/z 1447.80 [M + H]^+^), C16 (m/z 1461.82 [M + H]^+^), C17 (m/z 1475.84 [M + H]^+^), and C18 (m/z 1489.85 [M + H]^+^) (Figures 3E,F).

### Purification of YA215 lipopeptide extracts

**FIGURE 4:**
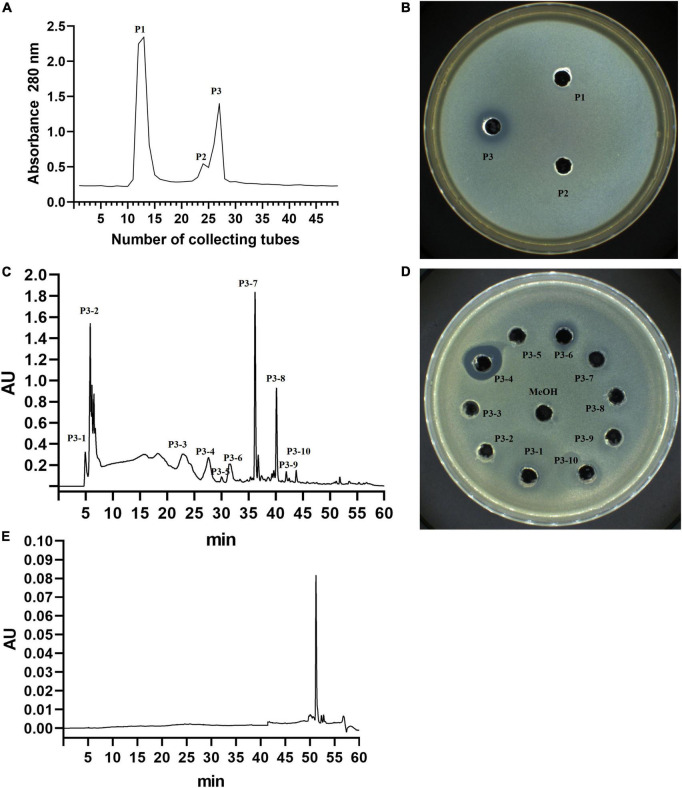
Isolation and purification of YA215 lipopeptide extracts (YA215LE). **(A)** Purification of YA215LE by gel column chromatography. **(B)** The bacteriostatic activity of the P3 fraction by gel column chromatography on *Escherichia coli*. **(C)** Purification of the P3 fraction by semipreparative reverse-phase high-performance liquid chromatography (RP-HPLC) using a C18 column. **(D)** The bacteriostatic activity of the P3-4 fraction by semipreparative RP-HPLC on *Escherichia coli*. **(E)** Determination of the purity of the P3-4 fraction by RP-HPLC.

First, three separation fractions were obtained from the separation of YA215LE by gel column chromatography (Figure 4A). After testing the antibacterial activity of the three separation fractions, the results showed that P3 exhibited good antibacterial activity (Figure 4B). The P3 fraction was further separated by semiprepared column liquid phase separation, and the results showed that the P3 component was more a complex with 10 components (Figure 4C). Among the separation fractions, the P3-4 fraction exhibited the highest antibacterial activity against *E. coli* (Figure 4D). This fraction was then was further checked by an analytical RP-HPLC system. The results showed that the P3-4 fraction was considered a pure product (Figure 4E) and was named BVYA1. BVYA1 was collected and concentrated, and further identified and analyzed by LC-MS/MS.

### Identification of the BVYA1 lipopeptide

**FIGURE 5:**
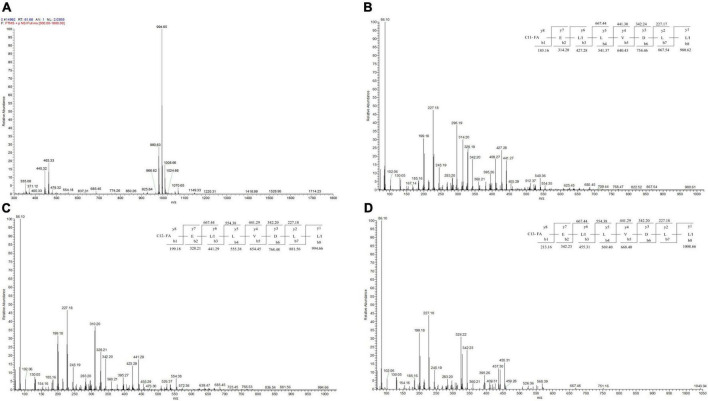
LC-MS/MS spectrum of the BVYA1 lipopeptide. **(A)** MS spectrum of the BVYA1 lipopeptide, **(B)** MS/MS spectra of [M + H]^+^ ions at m/z 980.62, **(C)** MS/MS spectra of [M + H]^+^ ions at m/z 994.66, **(D)** MS/MS spectra of [M + H]^+^ ions at m/z 1008.66.

LC-MS/MS analysis in the positive ionization mode was carried out to identify the BVYA1 lipopeptide. First, based on the results of the primary mass spectrometry, it can be concluded that the most abundant fragment ions in the positive ion mode were observed at m/z 994.66, while ions at m/z 980.62 and 1008.66 with a lower intensity were also observed (Figure 5A). The molecular ions in these peaks differed by 14 days from each other, which illustrated that the three components might be homologs. According to the mass numbers reported in the literatures (36–39), the parent ions at m/z 980.62, 994.66 and 1008.66 could be attributed to surfactins of the lipopeptides class. Second, MS/MS analysis was used to further analyze the parent ions at m/z 994.66, 980.62, and 1008.66. *De novo* peptide sequencing was performed by evaluating the *b*- and *y*-type consecutive ions. The parent ions at m/z 980.62, 994.66, and 1008.66 were found to contain the same peptide sequence, and their peptide sequences were identified as Glu–Leu/Ile–Leu–Val–Asp–Leu–Leu/Ile. In addition, the fatty acid moieties of the parent ions at m/z 980.62, 994.66, and 1008.66 comprised chains of different lengths from C11 to C13 (Figures 5B–D).

### The minimum inhibitory concentration of BVYA1

**FIGURE 6:**
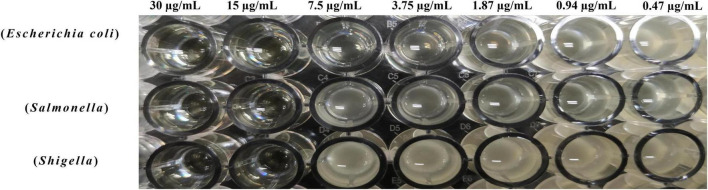
Determination of the minimum inhibitory concentration (MIC) of BVYA1 to indicator bacteria by the double dilution method.

The MIC of the purified BVYA1 was determined by a broth microdilution assay in a sterile 96-well microtiter plate. It can be seen from Figure 6, Table 1 show that the MICs of BVYA1 to *Escherichia coli*, *Salmonella*, and *Shigella* were 7.5l, 15, and 15 μg/ml, respectively. The results show that BVYA1 exhibits significant antibacterial activity.

**TABLE 1:** Results of minimum inhibitory concentration (MIC) of BVYA1 to indicator bacteria.

Indicator bacteria	MIC
*Escherichia coli*	7.5 μg/ml
*Salmonella*	15 μg/ml
*Shigella*	15 μg/ml

### Mechanism of the antibacterial action of BVYA1

#### Scanning electron microscopy assay

**FIGURE 7:**
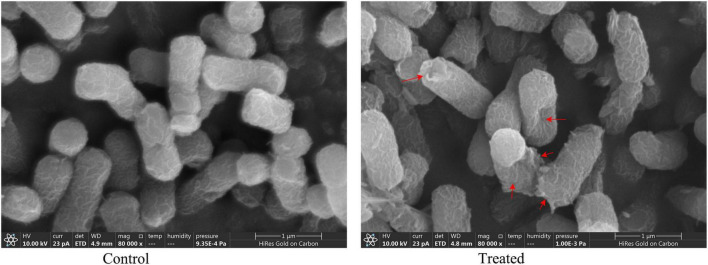
Morphological changes in *E. coli* treated with PBS and 2 × MIC BVYA1. Indentations in the cell membrane are indicated by red arrows.

To explore the antibacterial mechanism of BVYA1, the effect of BVYA1 on the surface morphology of *E. coli* was observed by SEM. As shown in Figure 7. The surface of *E. coli* cells treated with PBS was normal, smooth, and intact. However, the surface of *E. coli* cells treated with 2 × MIC BVYA1 was wrinkled and rough, and there were many obvious dents.

#### Bacterial membrane disruption activity assay

**FIGURE 8:**
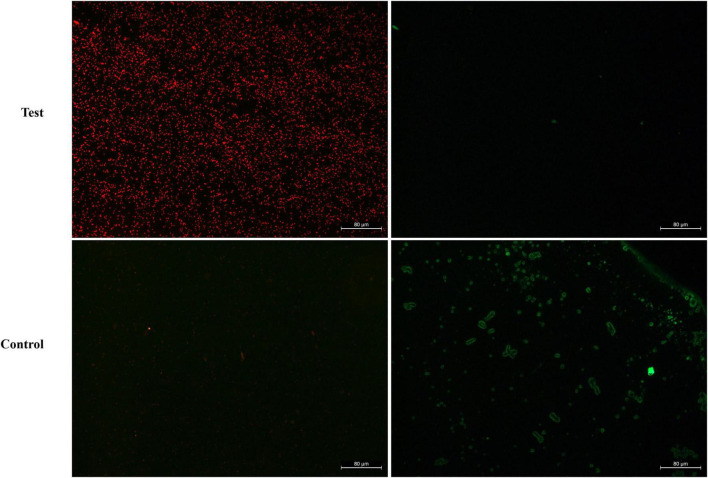
Fluorescence microscopy analysis of propidium iodide (PI) uptake during the membrane permeabilization assay. Control and treated represent fluorescent images of *E. coli* cells treated with PBS and 2 × MIC BVYA1, respectively.

To further understand the antibacterial mechanism of BVYA1, the destructive effect of BVYA1 on the membrane integrity of *E. coli* was evaluated by the calcein-AM/PI double-staining assay. Calcein-AM is a cell staining reagent that fluorescently labels live cells and fluoresces green (Ex = 490 nm, Em = 515 nm) when it enters the live cells. PI is an impermeable nucleic acid dye that only enters cells with damaged membranes. PI shows red fluorescence under laser excitation at 488 nm. Compared with the *E. coli* cells treated with PBS, the BVYA1 treatment group showed a large number of red PI in the *E. coli* cells, indicating that BVYA1 could penetrate the *E. coli* cell membrane (Figure 8). This result is consistent with the SEM results, suggesting that the mechanism of membrane destruction mediated by BVYA1 may be an vital reason for its antibacterial activity.

## Discussion

The increasing the burden and health risks of AMR pose a serious threat not only to public health but also to global food security, safety, and the economy. Due to the urgent necessity of protecting the environmental and achieving green and sustainable development, the search for a new and safe alternative to traditional antibiotics has become an urgent problem that must be solved with research. Lipopeptides exhibit a unique chemical composition and amphiphilic ring structure, which make their structure stable and can maintain high activity under some unfavorable conditions, such as high temperature, ultraviolet light and different pH values and proteases (12, 20). In addition, these lipopeptides exhibit unique properties, such as being easily biodegradable, eco-friendly, non-polluting biomolecules and safety (21, 22). With these advantages, lipopeptides are becoming increasingly vital in applications such as pharmaceuticals, agriculture and food preservation (21, 23). Therefore, lipopeptides have vast potential to become a novel and safe alternative to traditional antibiotics. Hence, in an effort to isolate and identify antibacterial lipopeptides as a novel and safe alternative to traditional antibiotics, this study was designed to isolate, purify, and characterize antibacterial lipopeptides from B. velezensis YA215 that was isolated from the mangrove area in Beibu Gulf, Guangxi, China.

The strain YA215, which was isolated from the mangrove area in Beibu Gulf, Guangxi, China, was identified as B. velezensis based on morphological and 16S rRNA gene sequences (Figure 1). Previous studies have demonstrated that B. velezensis inhibits the growth of various microorganisms (40–42). In this study, YA215LE extracted from the strain B. velezensis YA215 also exhibited strong bacteriostatic activity against gram-positive bacteria, such as *Bacillus licheniformis*, *Bacillus cereus*, *Staphylococcus aureus, Bacillus nanotuberculosis*, *Bacillus subtilis*, and *Bacillus subtilis*, and against gram-negative bacteria, such as *Escherichia coli*, *Salmonella typhimurium*, and *Shigella flexneri*. In addition, results of antifungal activity test showing that YA215LE exhibited stronger antifungal activities against *Fusarium oxysporum f.sp.cubense*, *Rhizopus stolonifer*, and *Penicillium expansum* (Figure 2). These results suggest that YA215LE exhibits significant broad-spectrum antimicrobial activity. Previous literature has demonstrated the following three main groups of lipopeptide substances act as bacterial inhibitors: surfactin, iturin, and fengycin (12, 35) In this research, LC-MS analysis was used to primarily identify the composition of YA215LE. The main antimicrobial compounds in YA215LE were identified as three kinds of lipopeptides, including homologs of surfactin, fengycin, and iturin (Figure 3). These results indicate that YA215LE extracted from strain B. velezensis YA215 may be used as a novel alternative to traditional antibiotics agents for combating several pathogens.

Surfactin is synthesized in a non-ribosomal manner in Bacillus. Multiple enzymes called non-ribosomal peptide synthases are involved (43). The surfactin structure consists of a cyclic peptide linked to a fatty acid moiety, which contains the heptapeptide sequence Glu–Leu–Leu–Val—Asp—Leu–Leu and the C12-C17 β-hydroxy fatty acid chain. The surfactin family contains several variants with different amino acid compositions (Ala, Val, Leu, or Ile amino acid variations at positions two, four, and seven) or with different lengths, branches, and acyl chain saturations (44). As previously reported in the literature, MS/MS analysis was used to identify the molecular structures of lipopeptides (38, 45). In the current study, after crude YA215LE was further isolated and purified, a component with significant antibacterial activity against indicator bacteria was screened and identified, and this component was composed of three surfactin homologs ([M + H]^+^: m/z 980.62; 994.66; 1008) (Figure 5A). Among them, 994.66 m/z was the main component, and the other two components were relatively low. MS/MS analysis was used to further analyze the parent ions at m/z 994.66, 980.62, and 1008.66. *De novo* peptide sequencing was performed by evaluating the *b*- and *y*- type consecutive ions. The parent ions at m/z 980.62, 994.66, and 1008.66 were found to have the same peptide sequence, and their peptide sequences were identified as Glu–Leu/Ile–Leu–Val–Asp–Leu–Leu/Ile. In addition, the fatty acid moieties of the parent ions at m/z 980.62, 994.66, and 1008.66 comprised chains of different lengths from C11 to C13 (Figures 5B–D). Interestingly, [M + H]^+^ at m/z 980.62 was detected for the first time in B. velezensis, which demonstrates that the strain corresponds to a new surfactin variant. The primary molecule of m/z 980.62 contains the heptapeptide sequence Glu–Leu/Ile–Leu–Val–Asp–Leu–Leu/Ile. and C11 β-hydroxy fatty acid chain.

Minimum inhibitory concentration assays were used to evaluate the antimicrobial activity of lipopeptides (19, 46). Previous studies have found that the MIC of surfactin is variable, which may be related to the diversity of its structure (8). Such as, Li et al. provided strong evidence that the MIC of the lipopeptide extract showed significant inhibitory effect on bacteria lipopeptide (19). Abdelli et al. found that lipopeptide presented a MIC value of 3.125 mg/ml against *E. coli* (47). In the current study, the MICs of BVYA1 to *E. coli*, *Salmonella* and *Shigella* were 7.5, 15, and 15 μg/ml, respectively (Figure 6 and Table 1), which further confirmed the remarkable inhibitory effect of BVYA1 lipopeptide on bacteria.

Bacillus exhibit great antimicrobial potential because they are able to produce lipopeptides that exhibit high activity against various microorganisms (48, 49). The structure of these lipopeptides are amphiphilic and therefore lipopeptides can interfere with the biofilm structure of microorganisms (50, 51). Previous research reported that lipopeptides achieved bactericidal effects by disrupting the permeability barrier and permeability of bacterial membranes (52, 53). In this study, the effect of BVYA1 on *E. coli* was observed using SEM and the calcein-AM/PI double-staining assay. Under SEM, the surface of *E. coli* cells treated with BVYA1 was wrinkled and rough, and there were many obvious dents (Figure 7). Under the calcein-AM/PI double-staining assay, when *E. coli* were treated with BVYA1, a large amount of red PI was found in the *E. coli* cells (Figure 8). These results suggest that BVYA1 lipopeptides may inhibit the growth of *E. coli* by effectively permeabilizing the cytoplasmic membrane and disrupting the morphology of targeted bacterial cells. However, the comprehensive mechanisms of the antibacterial effects of BVYA1 lipopeptide against *E. coli* need to be further investigated.

## Conclusion

We isolated a novel Bacillus velezensis strain from the mangrove area in Beibu Gulf, Guangxi, China, namely, B. velezensis YA215, which exhibits antibacterial activity against the indicator strains of gram-positive bacteria, gram-negative bacteria, and fungi. Three groups of lipopeptides (surfactin, iturin, and fengycin) produced by B. velezensis YA215 were identified, and the BVYA1 lipopeptide showed remarkable antibacterial activity, this activity may be associated with the effective permeabilization of the cytoplasmic membrane and disruption of the morphology of targeted bacterial cells. Our results also suggested that B. velezensis YA215 and its lipopeptides could be used as promising antibacterial agents against gram-positive bacteria, gram-negative bacteria, and fungi. In addition, YA215LE may become a novel alternative to traditional antibiotics from the B. velezensis YA215 strain, and these lipopeptides may have good prospects for application in food and agriculture.

## Data availability statement

The original contributions presented in this study are included in the article/supplementary material, further inquiries can be directed to the corresponding author.

## Author contributions

XL was the guarantor of the overall content, conceived the idea, and assessed each study. FY and XL drafted the study. YS and YQ performed the statistical analysis. HF and XP selected and retrieved relevant manuscript. All authors revised and approved the final manuscript.
